# Limited natural regeneration of unique Scalesia forest following invasive plant removal in Galapagos

**DOI:** 10.1371/journal.pone.0258467

**Published:** 2021-10-13

**Authors:** Anna Walentowitz, Michael Manthey, María Belén Bentet Preciado, Rafael Chango, Christian Sevilla, Heinke Jäger

**Affiliations:** 1 Department of Biogeography, University of Bayreuth, Bayreuth, Germany; 2 Institute of Botany and Landscape Ecology, University of Greifswald, Greifswald, Germany; 3 Universidad Agraria del Ecuador, Guayaquil, Ecuador; 4 Galapagos National Park Directorate, Galapagos, Ecuador; 5 Charles Darwin Research Station, Charles Darwin Foundation, Santa Cruz, Galapagos, Ecuador; Estacion Experimental de Zonas Aridas, SPAIN

## Abstract

More than 60% of the flora of the Galapagos Islands is introduced and some of these species have become invasive, severely altering ecosystems. An example of an affected ecosystem is the Scalesia forest, originally dominated by the endemic giant daisy tree *Scalesia pedunculata* (Asteraceae). The remnant patches of this unique forest are increasingly being invaded by introduced plants, mainly by *Rubus niveus* (blackberry, Rosaceae). To help large-scale restoration of this ecologically important forest, we seek to better understand the natural regeneration of *S*. *pedunculata* after invasive plant control. We monitored naturally recruited *S*. *pedunculata* saplings and young trees over five years in an area where invasive plant species are continuously being removed by manual means. We measured survival, height and growth of *S*. *pedunculata* saplings and young trees along permanent transects. Percent cover of surrounding plant species and of canopy shade directly above each *S*. *pedunculata* individual were determined, as well as distance to the next mature *S*. *pedunculata* tree. We identified potential factors influencing initial sapling survival and growth by applying generalized linear models. Results showed a rapid growth of saplings and young trees of up to 0.45 cm per day and a high mortality rate, as is typical for pioneer species like *S*. *pedunculata*. Sapling survival, growth and mortality seemed to be influenced by light availability, surrounding vegetation and distance to the next adult *S*. *pedunculata* tree. We concluded that natural regeneration of *S*. *pedunculata* was high only five months after the last herbicide application but that 95% of these recruits had died over the 5-year period. Further studies are needed to corroborate whether the number of surviving trees is sufficient to replace the aging adult trees and this way maintain remnants of the Scalesia forest. Urgent action is needed to help improve future restoration strategies to prevent further degradation of this rapidly shrinking threatened forest ecosystem.

## Introduction

As a direct consequence of human activity, island ecosystems worldwide are being transformed by invasive plant and animal species [1]. Many efforts are underway to control and eradicate invasives and restore island ecosystems, intending to conserve native and endemic species diversity [2]. Evaluating the success of these restoration projects is often constrained by a paucity of long-term ecological monitoring data [3, 4]. For example, the rate at which tropical forests recover from disturbance (e.g., deforestation, control of invasive species) can vary strongly, and understanding the factors driving the rate of recovery is critical to developing effective restoration measures [5]. Long-term observations of biotic and abiotic conditions within the area under restoration are indispensable to assessing project success [6, 7]. Several long-term restoration projects have been undertaken in the Galapagos Islands (e.g., mammal eradication [8]; plant eradication [9]), demonstrating that even in archipelagoes with comparatively late onset of human settlement like Galapagos [10], active ecological restoration is necessary to protect native ecosystems and conserve biodiversity.

There are about 810 introduced plant species in Galapagos [11] and some of these have become invasive, severely affecting the composition of plant communities [12]. A unique ecosystem under threat is the Scalesia forest, originally dominated by the endemic daisy tree *Scalesia pedunculata* (henceforth *S*. *pedunculata*) that occurs on four islands within the archipelago [13]. On Santa Cruz, the forest suffered massive reductions due to a history of deforestation by land use change and grazing and browsing by goats, pigs and donkeys [14, 15]. As a consequence, the remnant forest patches now comprise only 1% of the former distribution [16]. Species composition and population structure of *S*. *pedunculata* in these patches have been severely transformed by invasive plants, especially by *Rubus niveus* (Rosaceae) and *Cestrum auriculatum* (Solanaceae) [17, 18]. The high percent of *R*. *niveus* cover suppresses regeneration of *S*. *pedunculata* [18] and reduces the native species richness in the invaded areas [17]. On Santa Cruz, the remnant forest is considered a key ecosystem for many endemic bird and insect species, like the Darwin’s finches that forage and breed in the Scalesia forest [19, 20].

*Scalesia pedunculata* is a pioneer species that exhibits soft wood [21], fast growth and a short life cycle of 15–20 years [13, 14, 17, 21]. It used to dominate in a succession of life stages, with few trees of other species associated [14]. However, with shade-casting thickets of *R*. *niveus* in the understory, there is almost no germination of *S*. *pedunculata*, resulting in an extremely low natural regeneration of *S*. *pedunculata* [18]. This, combined with the high mortality rate of recruits, has led to a dramatic decline of the *S*. *pedunculata* population over the last decade [17, 18].

To address the increasing threat posed by *R*. *niveus*, the Galapagos National Park Directorate (GNPD) has been controlling this invasive species for over 20 years in different areas of the Galapagos National Park. A study by the authors carried out between 2014 and 2016 documented natural regeneration of *S*. *pedunculata* after invasive species control, but only over a short time period [18]. Therefore, in this study, we measured biotic parameters in 2015 and survival and growth of *S*. *pedunculata* saplings and trees from 2015 to 2020 in an experimental trial area of 6 ha in the Scalesia forest on Santa Cruz. By doing so, we sought to increase our understanding of the factors influencing regeneration and recruitment of *S*. *pedunculata*, following the removal of invasive plant species.

We hypothesized, that (1) the initial growth and mortality rates of *S*. *pedunculata* saplings and young trees in the remnant forest under restoration would be high, (2) the shading by the canopy would lead to higher sapling and young tree mortality and reduced growth rates, (3) sapling and young tree survival and growth rates would increase with distance from the next mature *S*. *pedunculata* tree, and (4) sapling and young tree survival and growth would decrease with higher cover of surrounding vegetation.

## Methods

### Study site

**Fig 1:**
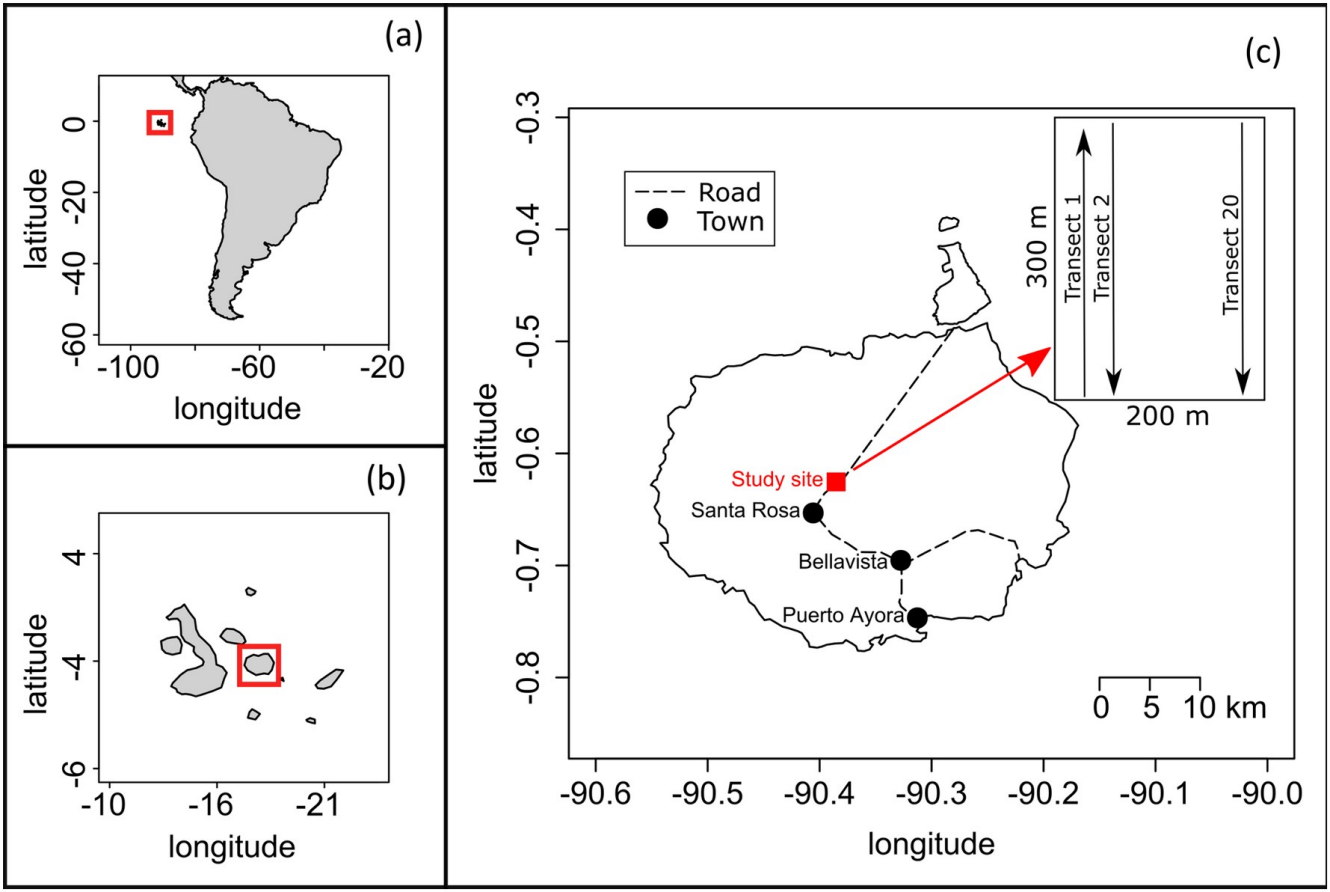
Location of the study site. (a) South America and the Galapagos Islands (red square). (b) Galapagos Islands with Santa Cruz (red square). (c) Santa Cruz with study site indicated in red and sketch of the sampling design. Projection: WGS 84 EPSG 4326.

The study was carried out in the Scalesia forest remnant in the highlands of Santa Cruz (Galapagos) at an altitude of about 400–550 m a.s.l. [13], near the twin volcanic sinkholes “Los Gemelos” (Fig 1). Mean annual precipitation during the five-year study period ranged from 736 mm in 2019 to 1244 mm in 2017, but the mean for all study years was lower than the long-term yearly average of 1380 mm (based on data from 1987 to 2019) (S1 Fig). Average daily temperature in the study area was 22.2°C [22]. The forest was composed of the endemic tree *Scalesia pedunculata* (Asteraceae, about 33% cover), accompanied by the shrubs *Tournefortia rufo-sericea* (Boraginaceae, endemic, about 6% cover), *Chiococca alba* (Rubiaceae, native, about 8% cover), *Psychotria rufipes* (Rubiaceae, endemic, about 3% cover) and *Zanthoxylum fagara* (Rutaceae, native, about 5% cover) [23, percent cover data Jäger, unpubl. data]. Invasive *R*. *niveus* formed dense thickets in the forest’s understory (about 64% cover) and other introduced and invasive shrubs, like *C*. *auriculatum* (about 18% cover) and *Psidium guajava* (Myrtaceae, 2% cover) co-occurred [17, 18]. These species, as well as the introduced and invasive herb *Tradescantia fluminensis* (Commelinaceae, about 36% cover), had been controlled by the GNPD in an experimental trial area of 6 ha since 2014. Initial control consisted of cutting the *R*. *niveus* and *C*. *auriculatum* bush to about 5 cm off the ground with a machete and spraying the regrowth with a combination of the herbicides Combo© and glyphosate after a month. This was repeated monthly two more times, with the last herbicide application occurring five months before the onset of our study in April 2015. Over the following five years, only manual control was carried out every three months (machete, weed trimmer, and hand-pulling) to prevent regrowth of invasive species, mainly of *R*. *niveus*.

### Data sampling and analysis

Field work in the Scalesia forest was conducted under permits issued by the Galapagos National Park Directorate (PC-19-15, PC-50-16, PC-42-17, PC-50-18, PC-55-19 and PC-26-20). Natural regeneration of *S*. *pedunculata* was determined by establishing 20 parallel permanent transects of 300 m, 10 m apart, in a S-SE to N-NW direction within the study area (Fig 1c). All *S*. *pedunculata* saplings of a height up to 100 cm (assuming that these had emerged after the last herbicide application 5 months prior to the onset of this study), growing within 1 m to both sides of these transects, were marked with aluminum tags and the exact location was measured with a handheld GPS device (Garmin GPSMAP 65 Series). Measurements were first recorded in April 2015 and then repeated seven times over the course of five years, according to time availability: in June 2015, February 2016, August 2016, March 2017, January 2018, April 2019 and March 2020. At each monitoring event, we documented survival of the marked individuals and calculated mortality rates. Saplings were determined as dead if they were entirely brown and mostly detached from the ground or partly decomposed. We also measured the height of saplings and young trees and calculated mean and maximum growth (defined as the change in height). Daily growth was calculated at the end of the study period by dividing total growth over the five years by the number of days between the first and last monitoring event. Data were processed and descriptive and inferential statistics applied in R Version 4.0.0 [24].

Percent shading by canopy, distance to the next mature S. *pedunculata* tree and percent surrounding vegetation were only measured once at project start in April 2015 and were not repeated over the study period due to time constraints. For this, a photo of the forest canopy parallel to the ground over the top of each *S*. *pedunculata* individual, with a Nikon D3200 camera (Settings: 300 dpi; aperture value F/7.1; shutter speed 1/2000 sec.). The photos were converted into black and white images by maximizing the contrast in GIMP 2.8.22 [25]. Percent canopy cover (= shade) was calculated for each *S*. *pedunculata* sapling as the ratio between black and white pixels. Mean percent canopy shade and standard deviation were calculated for the total of all surviving and dead saplings. Distance of each sapling to the nearest mature *S*. *pedunculata* tree was measured in m. To determine percent cover of individual ground-covering plant species, the area of the saplings’ crown was projected onto the ground and defined as 100% cover, and cover of each species within this area was estimated as a fraction. For model building, the surrounding vegetation ground cover underneath each *S*. *pedunculata* sapling was calculated as the sum of the cover of all species. To determine factors influencing *S*. *pedunculata* sapling survival and growth during the first year, we implemented general linearized models (GLM) with binomial distribution and logit link function (for survival) and Gaussian distribution (for growth), with fitted curves at a 95% confidence interval. We conducted a correlation analysis of all biotic parameters (S2 Fig) with the package “corrplot” [26], using Pearson’s correlation coefficient. The GLMs for *S*. *pedunculata* sapling survival and growth were built considering the total percent surrounding vegetation cover.

## Results

### Survival

**Fig 2:**
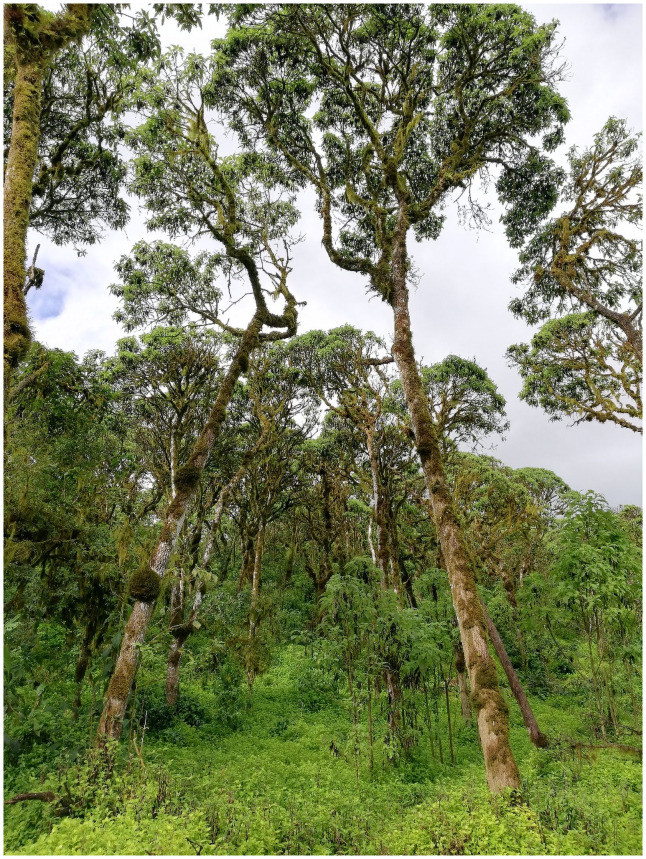
Mature *Scalesia pedunculata* trees in 2019 with saplings and young trees in the understory in a 6 ha study area in which invasive plant species, especially *Rubus niveus*, had continuously been removed by the Galapagos National Park Directory since 2014. The plant cover on the forest floor mainly consisted of the invasive carpet-forming *Tradescantia fluminensis*.

Five months after the last herbicide application, there was an abundant regeneration of *Scalesia pedunculata* in the study area of the Scalesia forest, but only very few were still alive towards the end of the study period (Fig 2).

**Fig 3:**
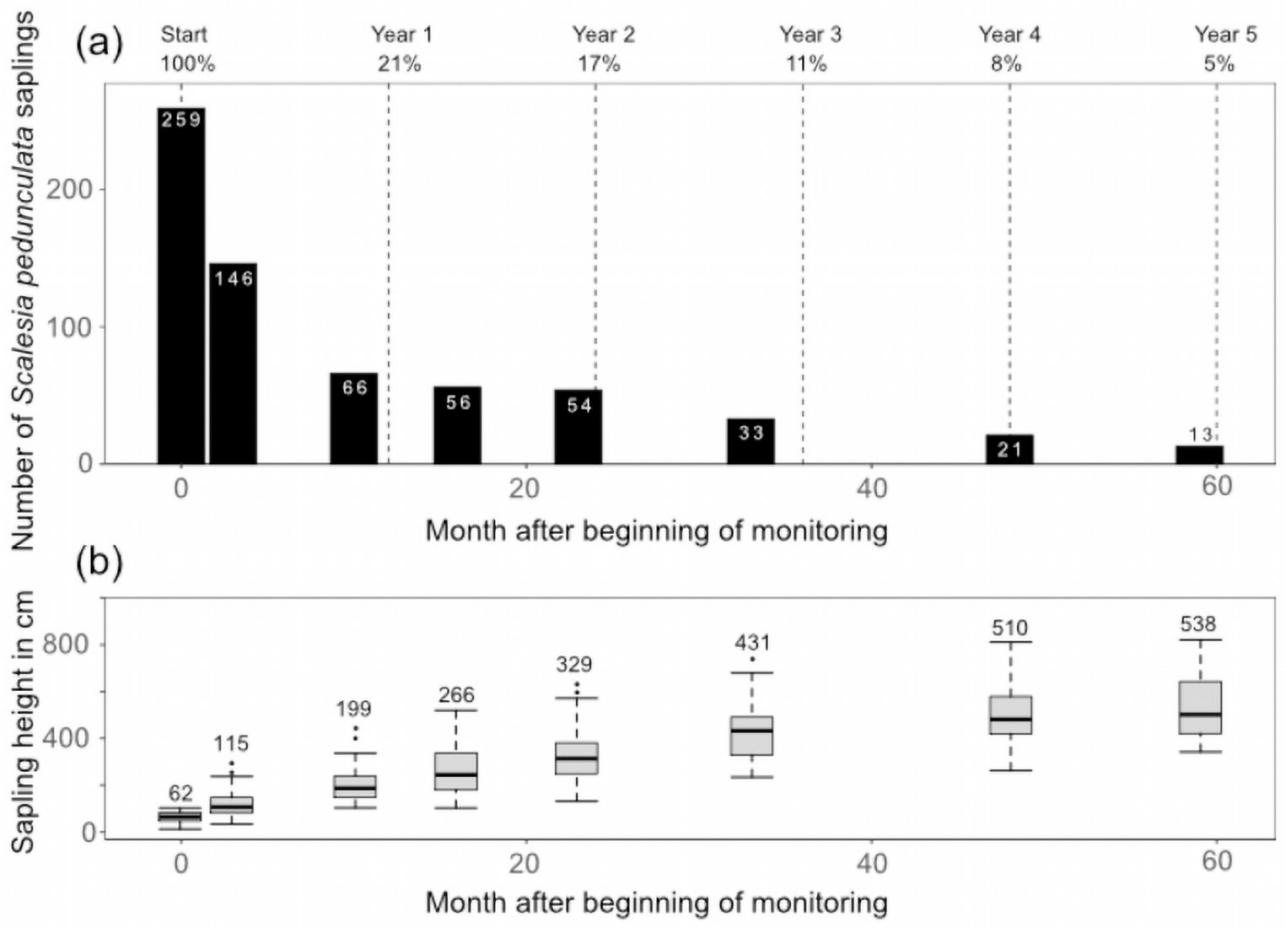
(a) Number (within bars) and percent (top row) of surviving *S*. *pedunculata* saplings and young trees over 5 years. The mortality rate over five years was 95%. (b) Height of the *S*. *pedunculata* saplings and young trees over time. The boxplots display the median and the interquartile range (25^th^ to 75^th^ percentile), whiskers indicate the variability outside upper and lower quartiles and outliers are displayed. Mean height is given on top of the boxplots.

Of the initial 259 saplings recorded in April 2015, only 13 individuals (5%) were found alive in 2020 (Fig 3a). The largest loss of individuals was recorded after the first year, with only 66 saplings still alive (21%, taking only those individuals into account that could be relocated) in February 2016. In another study in the same area, a similar high mortality rate of *S*. *pedunculata* recruits was observed (Jäger, unpubl. data). A total of 55 individuals could not be re-located, despite the fact that they were marked with an aluminum tag and their GPS location was known. These had most likely died or were trampled by the ongoing manual invasive plant control actions. Due to the rapid turn-over of organic material in the study area, aluminum tags were probably covered by this and therefore could not be found.

**Fig 4:**
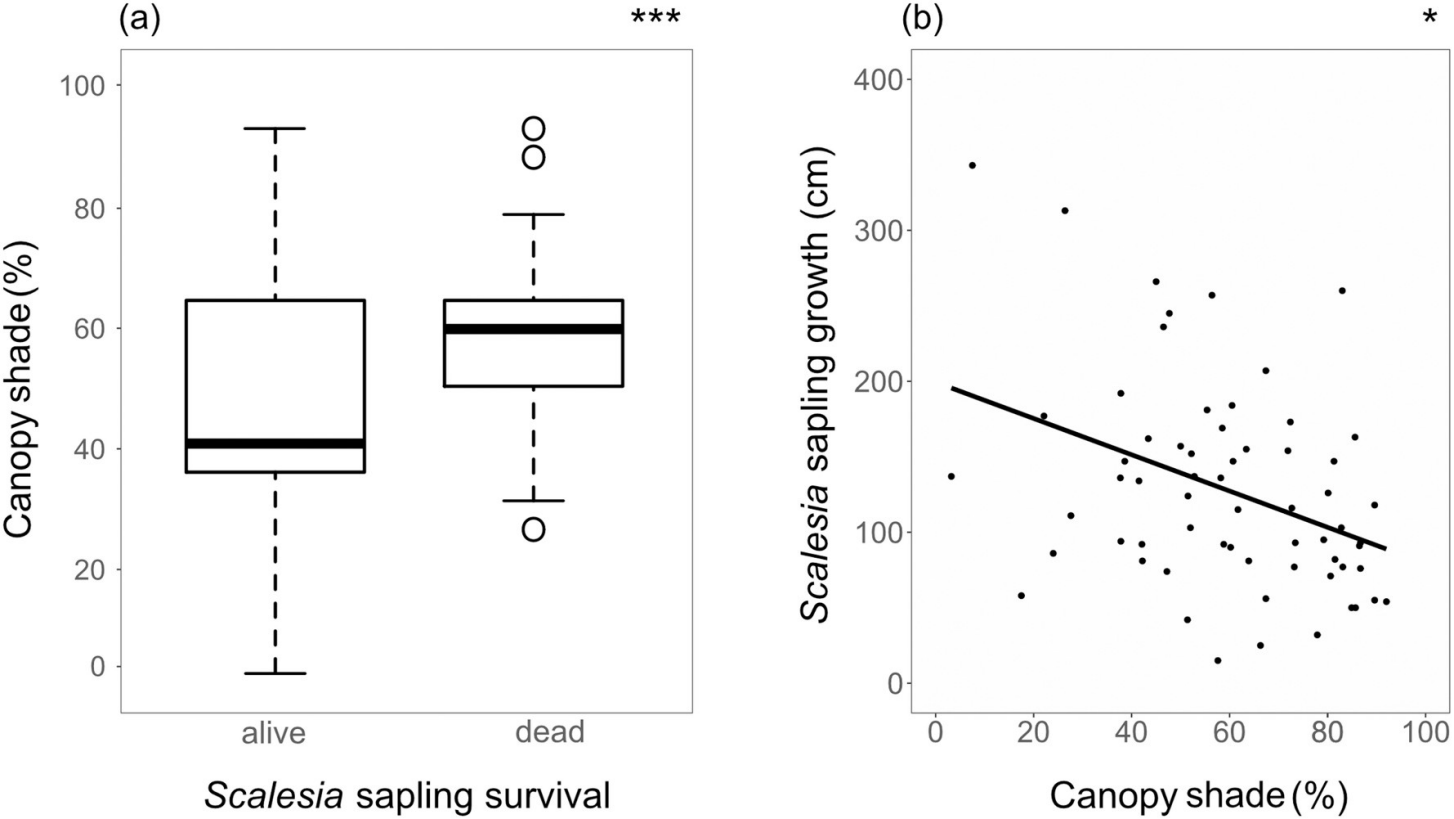
Percent canopy shade was significant in explaining *S*. *pedunculata* saplings mortality (a, GLM with binomial distribution and logit link function) and growth (b, GLM with a Gaussian distribution) after the first year. Boxplots (a) display the median, interquartile range (25^th^ to 75^th^ percentile), variability outside upper and lower quartiles (whiskers) and outliers. Significance levels are reported as: [*] p < 0.05; [***] p < 0.001.

Percent canopy shade had the highest explanatory power for sapling survival (p < 0.001) based on GLMs (Table 1 and Fig 4a). Saplings that were dead after the first year had been exposed to a higher percent total canopy shade than the ones that survived (72.2% ± 13 vs. 56.7% ± 21). The most dominant species in the canopy shade for both groups was *S*. *pedunculata*, followed by *C*. *auriculatum*. The distance of saplings to the next mature *S*. *pedunculata* was significantly larger for surviving saplings compared to dead saplings (p = 0.05, 2.13 m ± 1.05 vs 1.58 m, ± 0.81). Percent cover of the surrounding vegetation was higher for surviving saplings compared to dead saplings after the first year (52.8%, ± 35 vs. 41.7%, ± 29), but not significantly so (p = 0.09). This vegetation consisted mainly of *R*. *niveus* (5%) and *T*. *fluminensis* (25.9%). About 28.9% of the surviving saplings were shaded by *C*. *auriculatum* (presence/absence, not cover) at first monitoring in April 2015. Percent cover of all species is shown in S1 Table.

**Table 1:** *Scalesia pedunculata* sapling and young tree mortality was significantly correlated with percent canopy shade and distance to the next *S*. *pedunculata* tree, while percent cover of surrounding vegetation did not have a significant explanatory power.

Dependent: Sapling survival	Surviving saplings	Dead saplings	p
**Canopy shade (%)**	56.7 (21)	71.2 (13)	< 0.001
**Distance to next *S*. *pedunculata* tree (m)**	2.13 (1.05)	1.58 (0.81)	0.05
**Cover of surrounding vegetation (%)**	52.8 (35)	41.7 (29)	n.s.

Means are given for alive and dead saplings after the first year (standard deviation in parenthesis). P-values are based on a GLM (binomial distribution, logit function) with the three parameters ‘canopy shade’, ‘distance to the next *S*. *pedunculata*’ and ‘cover of surrounding vegetation’ included as explanatory variables.

### Height and growth

Average sapling height increased from 62 cm (±23) in 2015 to an average tree height of 538 cm (±170) in 2020 (Fig 3b), which amounts to a yearly average growth of 95 cm, with growth rates differing between years and individuals (S2 Table). Average daily growth over five years was 0.25 cm, but we also measured an average of 0.45 cm a day for the largest plant that reached a height of 820 cm in 2020.

Percent canopy shade was significantly negatively correlated with sapling growth (p < 0.05). Distance to the next mature *S*. *pedunculata* tree and percent surrounding vegetation cover were not correlated with sapling height (p < 0.05).

## Discussion

Our results showed that while control of *Rubus niveus* in the Scalesia forest on Santa Cruz facilitated an abundant natural regeneration of *Scalesia pedunculata*, only 5% of these new plants survived the 5-year study period (13 out of 259 individuals). A previous publication from the same study area reported a spectacular regeneration of *S*. *pedunculata* in 2016, but this was about 14 months after the last herbicide application, and no follow-up survey of the population was included [18]. The observed mortality rate of *S*. *pedunculata* saplings in our study might have been influenced by the last glyphosate application five months prior to the onset of the study, but it is not clear whether glyphosate residuals in the soil affect emerging seedlings [27]. Independently of this, high mortality rates had been observed in other studies addressing the life cycle of *S*. *pedunculata* and is typical for a pioneer species like *S*. *pedunculata* [13, 16, 21, 28]. Self-thinning could be a potential explanation since dead stems of *S*. *pedunculata* were found to be smaller than those of live individuals [29]. Although survival rates of *S*. *pedunculata* were low, it should be consider that this study followed only one cohort of trees that had emerged after the initial invasive plant species control. Recruits from the seed bank from subsequent years could have substituted those individuals from the first cohort monitored that had died off, as was observed by a study in the same area (Jäger, unpubl. data).

The *S*. *pedunculata* saplings in our study quickly grew into young trees, reaching a maximum height of 820 cm after five years, growing an average 0.45 cm a day. This finding is consistent with a study carried out in the 1970s, where trees reached 7–8 m in height after 3.5 to 5.5 years, but this was before invasive plants became a problem [28]. Percent shade and proximity to the next mature *S*. *pedunculata* tree had the highest explanatory power for initial sapling mortality and percent shade for a reduced sapling growth.

Besides the pioneer character and control actions, biotic factors influencing the initial mortality rate and growth of *S*. *pedunculata* saplings are important for Scalesia forest restoration. As hypothesized, we found that survivorship and height of *S*. *pedunculata* saplings correlated strongly with light availability. Overall, surviving saplings received less shade, which mainly stemmed from mature *S*. *pedunculata* trees or the invasive shrub *C*. *auriculatum*. We, therefore, confirmed the results of previous studies that reported a high light dependency of *S*. *pedunculata* [13, 14, 21]. In our study, invasive plant removal caused higher light availability, which in turn facilitated *S*. *pedunculata* regeneration. Previous studies reported massive dieback of mature *S*. *pedunculata* trees after the extreme 1982/83 El Niño event that created suitable conditions for natural *S*. *pedunculata* regeneration from seeds [13, 21]. In contrast to these studies, mature *S*. *pedunculata* trees were still present in our study area and the proximity to the nearest mature *S*. *pedunculata* tree was negatively associated with the survival of saplings. As *S*. *pedunculata* is the dominant tree species within this forest type [13], canopy shading is logically reduced at greater distances from mature trees. This aligns with the Janzen Connell hypothesis, which states that propagule survival is dependent on the distance to its parent tree [30, 31]. The distribution and survival of *S*. *pedunculata* seedlings thus seem to be partly the result of a trade-off between light availability, caused by distance to the shading canopy of the parent tree [32], and a short dispersal distance reported for this species [33].

Contrary to our hypothesis, higher vegetation cover surrounding *S*. *pedunculata* individuals did not significantly affect survival and growth rates of saplings. About 25.9% of this surrounding vegetation was composed of the invasive ground-covering plant *Tradescantia fluminensis*, and cover of this species increased significantly after control of *R*. *niveus* in the study area [18]. *Tradescantia fluminensis* is known to be a severe invader elsewhere [34] and has been shown to alter nutrient availability in temperate forests and to hinder native forest regeneration [35, 36]. Our study was carried out during an exceptionally dry period [22], which could have affected the natural *S*. *pedunculata* regeneration but our data do not allow us to determine whether co-occurrence of *T*. *fluminensis* and *S*. *pedunculata* is due to favorable microclimate or to facilitation or competition [37]. Thus, future research is needed to disentangle the influence of biotic factors from weather conditions on *S*. *pedunculata* regeneration over a longer study period. With an anticipated increase in frequency of El Niño (ENSO) events [38, 39], regeneration of *S*. *pedunculata* might differ significantly between years. The twofold pressure from unknown impacts caused by climate change (e.g., increasing temperatures, increasing precipitation and weather extremes) and the anticipated increase of species’ invasions, should be taken into account for future restoration actions in the Scalesia forest remnants [40].

In conclusion, invasive plant species have severely altered the Scalesia forest on Santa Cruz to a high degree [18, 41]. In combination with its historically reduced range (only 1% of the original forest distribution remains) [16], urgent restoration actions are needed. Our results indicate that natural regeneration of *S*. *pedunculata* is facilitated by invasive plant species removal. Due to the high mortality rate of recruited *S*. *pedunculata* saplings (95%), future restoration actions should include the planting of nursery-grown *S*. *pedunculata* seedlings and young trees, which has proven successful elsewhere [42]. The Scalesia forest is not only unique due to the endemic *S*. *pedunculata*, it is also an important ecosystem for associated (and endemic) invertebrate and bird species, like the Darwin’s finches [19, 20]. Although the *R*. *niveus* control had a temporary negative effect on the microhabitat use and feeding behavior of *Certhidea olivacea* (green warbler finch) and *Camarhynchus parvulus* (small tree finch), we call for urgent actions to remove *R*. *niveus* at a large scale to preserve the last Scalesia forest remnants on Santa Cruz [18, 20].

## Supporting information

S1 Fig Annual precipitation in the highlands of Santa Cruz Island before from 1989 to 2019 and during the study period indicated by black bars. (TIF)

S2 Fig Correlation plot of biotic factors of *S*. *pedunculata* sapling survival and growth based on Pearson’s correlation coefficient. Species names are abbreviated by taking the first three letters of their genus and epithet. RubNiv, TraFlu and veg refer to sapling’s surrounding vegetation while CesAur and ScaPed belong to the shading canopy.
(TIF)

S1 Table Initial surrounding vegetation (% cover of single species) of *S*. *pedunculata* saplings dead or alive after one year with standard deviation in parenthesis (SD). (DOCX)

S2 Table Average and maximum growth of *S*. *pedunculata* saplings and young trees over time. (DOCX)

S1 Data (CSV)

## References

[1] Russell JC, Kueffer C. Island Biodiversity in the Anthropocene. Annu. Rev. Environ. Resour. 2019;44: 31–60.

[2] Glen AS, Atkinson R, Campbell KJ, Hagen E, Holmes ND, Keitt BS, et al. Eradicating multiple invasive species on inhabited islands: the next big step in island restoration? Biol. Invasions 2013;15: 2589–2603.

[3] Suding KN. Toward an era of restoration in ecology: successes, failures and opportunities ahead. Annu. Rev. Ecol. Evol. Syst. 2011;42: 465–487.

[4] Nilsson C, Aradottir AL, Hagen D, Halldórsson G, Høegh K, Mitchell RJ, et al. Evaluating the process of ecological restoration. Ecol. Soc. 2016;21: 41.

[5] Holl KD. Restoration of tropical forests. In: van Andel J, Aronson J, editors. Restoration Ecology. West Sussex, United Kingdom: Wiley-Blackwell, Oxford & West Sussex; 2012. pp. 130–114.

[6] Wortley L, Hero JM, Howes M. Evaluating ecological restoration success: a review of the literature. Restor. Ecol. 21;2013: 537–543.

[7] Prior KM, Adams DC, Klepzig KD, Hulcr J. When does invasive species removal lead to ecological recovery? Implications for management success. Biol. Invasions 2018;20: 267–283.

[8] Carrion V, Donlan CJ, Campbell KJ, Lavoie C, Cruz F. Wide Island Restoration in the Galápagos Islands: Reducing costs of invasive mammal eradication programs and reinvasion risk. PLOS ONE 2011;6: e18835. 21589656 10.1371/journal.pone.0018835 PMC3092746

[9] Gardener MR, Trueman M, Buddenhagen C, Heleno R, Jäger H, Atkinson R, et al. A pragmatic approach to the management of plant invasions in Galapagos. In: Foxcroft LC, Pyšek P, Richardson DM, Genovesi P, editors. Plant invasions in protected areas: patterns, problems and challenges. Heidelberg, Germany: Springer; 2013. pp. 349–374.

[10] Machado A. An index of naturalness. J. Nat. Conserv. 2004;12: 95–110.

[11] Toral-Granda MV, Causton CE, Jäger H, Trueman M, Izurieta JC, Araujo E, et al. Alien species pathways to the Galapagos Islands, Ecuador. PLOS ONE 2017;12: 1–21. doi: 10.1371/journal.pone.0184379 28902860 PMC5597199

[12] Jäger H, Kowarik I, Tye A. Destruction without extinction: Long-term impacts of an invasive tree species on Galápagos highland vegetation. J. Ecology 2009;97, 1252–1263.

[13] Hamann O. Demographic studies of three indigenous stand-forming plant taxa (Scalesia, Opuntia, and Bursera) in the Gálapagos Islands, Ecuador. Biodivers. Conserv. 2001;10: 223–250.

[14] Itow S. Phytogeography and ecology of Scalesia (Compositae) endemic to the Galápagos Islands. Pac. Sci. 1995;49: 17–30.

[15] Lundh J. The farm area and cultivated plants on Santa Cruz, 1932–1965, with remarks on other parts of Galapagos. Galapagos Research 2006;64: 12–25.

[16] Mauchamp A, Atkinson R. Rapid, recent and irreversible habitat loss: Scalesia forest in the Galapagos Islands. Galapagos Report 2009–2010. GNPD, GCREG, CDF and GC. Puerto Ayora, Galápagos, Ecuador 2010: 108–112.

[17] Rentería JL, Gardener MR, Panetta FD, Atkinson R, Crawley MJ. Possible impacts of the invasive plant Rubus niveus on the native vegetation of the Scalesia forest in the Galapagos Islands. PLOS ONE 2012;10: 1–9. doi: 10.1371/journal.pone.0048106 23118934 PMC3485296

[18] Jäger H, Buchholz S, Cimadom A, Tebbich S, Rodríguez J, Barrera D, et al. Restoration of the blackberry-invaded Scalesia forest: Impacts on vegetation, invertebrates, and birds. Galapagos Report 2015–2016. GNPD, GCREG, CDF and GC. Puerto Ayora, Galápagos, Ecuador 2017: 145–149.

[19] Filek N, Cimadom A, Schulze CH, Jäger H, Tebbich S. The impact of invasive plant management on the foraging ecology of the Warbler Finch (Certhidea olivacea) and the Small Tree Finch (Camarhynchus parvulus) on Galápagos. J. Ornithol. 2017;159: 129–140. doi: 10.1007/s10336-017-1481-4 31998596 PMC6956869

[20] Cimadom A, Jäger H, Schulze CH, Hood-Nowotny R, Wappl C, Tebbich S. Weed management increases the detrimental effect of an invasive parasite on arboreal Darwin’s finches. Biol. Conserv. 2019;233: 93–101.

[21] Lawesson JE. Stand-level dieback and regeneration of forests in the Galápagos Islands. Vegetatio 1988;77: 87–93.

[22] Charles Darwin Foundation. Climatology Database Bellavista. 2020 [Cited 2020 August 20]. https://www.darwinfoundation.org/en/datazone/climate/bellavista.

[23] Eliasson U. Native climate forests. In: Perry R, editor. Key environments Galápagos. Oxford, UK: Pergamon Press; 1984. pp. 101–114.

[24] R Core Team. R: A language and environment for statistical computing. R Foundation for Statistical Computing. 2020. Vienna, Austria.

[25] GNU Image Manipulation Program, GIMP Version 2.8.22. 2017. https://www.gimp.org/.

[26] Wei T, Simko V. R package "corrplot": Visualization of a Correlation Matrix (Version 0.84). 2017. https://github.com/taiyun/corrplot.

[27] Helander M, Pauna A, Saikkonen K, et al. Glyphosate residues in soil affect crop plant germination and growth. Sci Rep. 2019;9: 19653. doi: 10.1038/s41598-019-56195-3 31873174 PMC6927981

[28] Hamann O. Dynamics of a stand of Scalesia pedunculata Hooker fil., Santa Cruz Island, Galapagos. Bot. J. Linn. Soc. 1979;78: 67–84.

[29] Runkle JR, Runkle WA. Structure and development of a Scalesia pedunculata stand in the Galapagos Islands. Galapagos Research 2005;63: 12–15.

[30] Janzen DH. Herbivores and the number of tree species in tropical forests. Am. Nat. 1970;104: 501–528.

[31] Connell JH. On the role of natural enemies in preventing competitive exclusion in some marine animals and in forest trees. In: Boer PJ, Gradwell GR, editors. Dynamics of Populations. Wageningen, Netherlands: Centre for Agricultural Publishing and Documentation; 1971. pp 298–312.

[32] Kobe RK. Vriesendorp CF. Conspecific density dependence in seedlings varies with species shade tolerance in a wet tropical forest. Ecol. Lett. 2011; 14: 503–510. doi: 10.1111/j.1461-0248.2011.01612.x 21429063

[33] Shimizu Y. Competitive relationships between tree species of Scalesia (S. pedunculata, S. cordata, S. microcephala) and introduced plants (Cinchona succirubra, Psidium guajava, Lantana camara) with reference to regeneration mechanism of Scalesia forests in the Galápagos Islands. Regional Views 1997;11: 23–172.

[34] CABI. Tradescantia fluminensis. In: Invasive Species Compendium. Wallingford, UK: CAB International. 2020. www.cabi.org/isc.

[35] Kelly D, Skipworth JP. Tradescantia fluminensis in a Manawatu (New Zealand) forest: I. Growth and effects on regeneration. N. Z. J. Bot. 1984;22: 393–397

[36] Standish RJ, Williams PA, Robertson AW, Scott NA, Hedderley DI. Invasion by a perennial herb increases decomposition rate and alters nutrient availability in warm temperate lowland forest remnants. Biol. Invasions 2004;6: 71–81.

[37] Steinbauer MJ, Beierkuhnlein C, Arfin Khan MAS, Harter DEV, Irl SDH, Jentsch A, et al. How to differentiate facilitation and environmentally driven co-existence. Appl. Veg. Sci. 2016;27: 1071–107.

[38] Trueman M, Hannah L, d’Ozouville N, Larrea I, Di Carlo G. Terrestrial ecosystems in Galapagos: Potential responses to climate change. Climate change vulnerabilitly assessment of the Galapagos Islands. WWF and Conservation International, USA, 2010: 29–46.

[39] Cai W, Borlace S, Lengaigne M, van Rensch P, Collins M, Vecchi G, et al. Increasing frequency of extreme El Niño events due to greenhouse warming. Nat. Clim. Change 2013;4: 111–116.

[40] Harris JA, Hobbs RJ, Higgs E, Aronson J. Ecological restoration and global climate change. Restor. Ecol. 2006;14: 170–176.

[41] Rivas-Torres G, Flory SL, Loiselle B. Plant community composition and structural characteristics of an invaded forest in the Galápagos. Biodivers. Conserv. 2018;27: 329–344.

[42] Holl KD, Aide TM. When and where to actively restore ecosystems? For. Ecol. Manag. 2011;261: 1558–1563.

