# Supplementary figures and images for: Limited natural regeneration of unique Scalesia forest following invasive plant removal in Galapagos
Source: PLoS One. 2021 Oct 13;16(10):e0258467. doi: 10.1371/journal.pone.0258467 (PMC8513895; doi:10.1371/journal.pone.0258467)

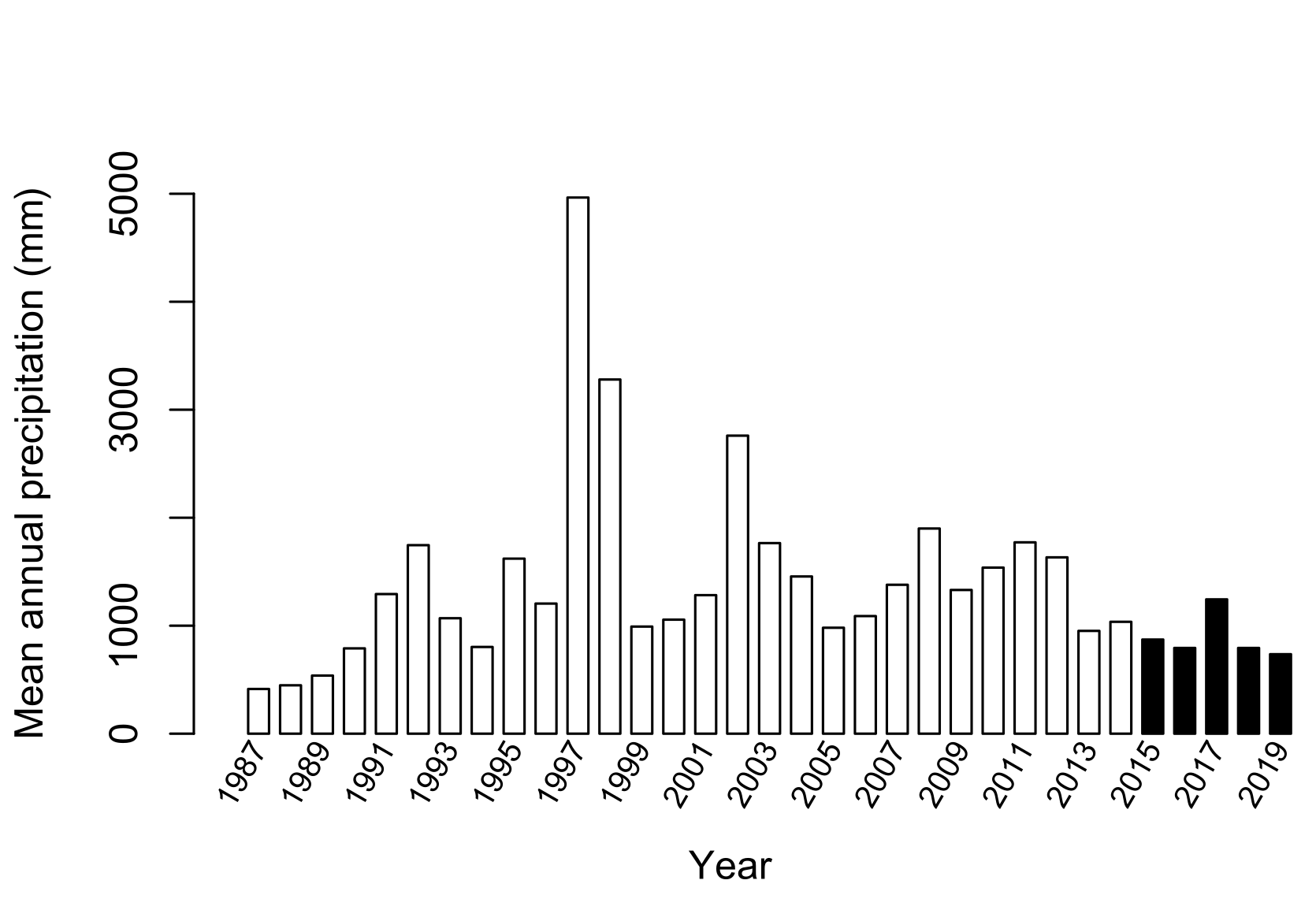

Supplement: S1 Fig — (TIF) [file pone.0258467.s001.tif]

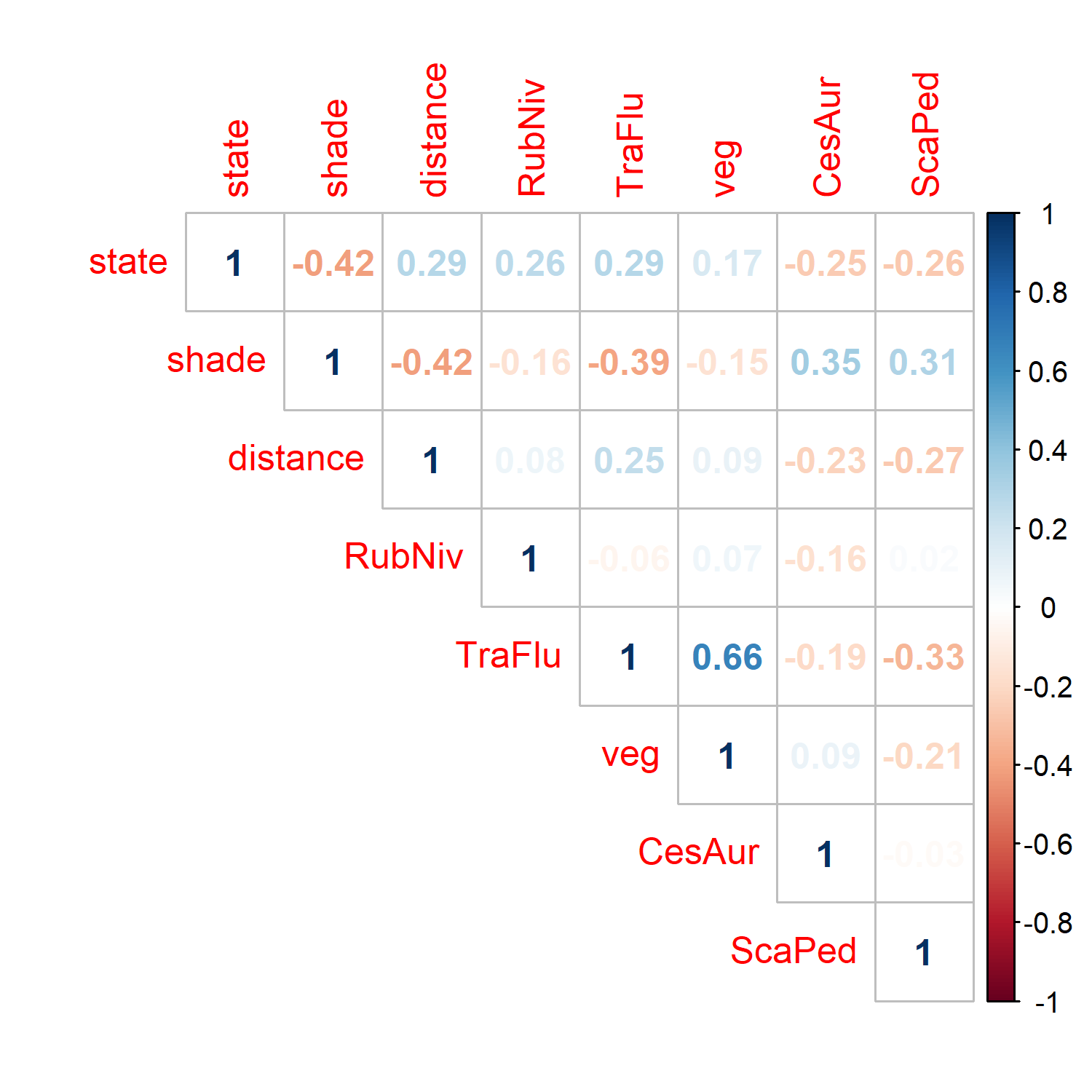

Supplement: S2 Fig — Species names are abbreviated by taking the first three letters of their genus and epithet. RubNiv, TraFlu and veg refer to sapling’s surrounding vegetation while CesAur and ScaPed belong to the shading canopy. (TIF) [file pone.0258467.s002.tif]
