# Supplementary material for: Limited natural regeneration of unique Scalesia forest following invasive plant removal in Galapagos
Source: PLoS One. 2021 Oct 13;16(10):e0258467. doi: 10.1371/journal.pone.0258467 (PMC8513895; doi:10.1371/journal.pone.0258467)
Supplement: S1 Table — (DOCX) [file pone.0258467.s003.docx]

**S3 Table:** Initial surrounding vegetation (% cover of single species) of *S. pedunculata* saplings dead or alive after one year with standard deviation in parenthesis (SD).

| **Species** | **% cover surrounding alive seedlings (SD)** | **% cover surrounding dead seedlings (SD)** |
| --- | --- | --- |
| *Asplenium auritum* Sw. | 0.6 (2.9) | 0.5 (4.1) |
| *Asplenium cristatum* Lam. | 0.1 (1.1) | 0 |
| *Blechnum occidentale* L. | 0.6 (3.3) | 0.2 (1.3) |
| *Blechnum polypodioides* Raddi | 0.4 (2.4) | 0 |
| *Campyloneurum phyllitidis* (L.) C. Presl | 1.3 (5.6) | 0.8 (5.3) |
| *Cestrum auriculatum* L’Hér. | 3.3 (8.7) | 11.3 (15.6) |
| *Chiococca alba* (L.) Hitchc. | 0.5 (3.5) | 0 |
| *Commelina diffusa* Burm. f. | 0.7 (4.6) | 0.2 (1.8) |
| *Conyza bonariensis* (L.) Cronquist | 0.1 (1.1) | 0.4 (4.6) |
| *Doryopteris pedata var. palmata* (Willd) Hicken | 0.2 (1.5) | 0.1 (0.9) |
| *Galium galapagoense* Wiggins | 0 | 0.4 (3.8) |
| *Ichnanthus nemorosus* (Sw.) Döll | 1.2 (5.7) | 0.9 (3.9) |
| *Paspalum conjugatum* Bergius | 1.1 (6.8) | 0.1 (0.9) |
| *Passiflora colinvauxii* Wiggins | 0 | 0.7 (7.3) |
| *Peperomia inaequalifolia* Ruiz & Pav. | 0.4 (2.4) | 0.2 (2.0) |
| *Pilea baurii* Robinson | 3.6 (10.3) | 3.7 (8.9) |
| *Psidium guajava* L. | 0.4 (3.3) | 0 |
| *Psychotria rufipes* Hook. f. | 0 | 0.2 (1.3) |
| *Pteris quadriauriata* Retz. | 0.1 (1.1) | 0.1 (0.9) |
| *Rubus niveus* Thunb. | 5.1 (11.7) | 0.5 (2.5) |
| *Scalesia pedunculata* Hook. f. | 1.2 (4.2) | 0 |
| *Sida rhombifolia* L. | 0 | 0.1 (0.9) |
| *Solanum americanum* Mill. | 0.6 (2.9) | 0.8 (4.9) |
| *Tetramerium nervosum* Nees | 2.9 (9) | 10.9 (14.8) |
| *Thelypteris conspersa* (Schrad.) A.R.Sm. | 0.2 (2.2) | 0 |
| *Thelypteris hispidula* (Decne.) C.F. Reed | 0 | 0.1 (0.9) |
| *Thelypteris tetragona* (Sw.) Small | 0.6 (2.4) | 0 |
| *Tournefortia rufo-sericea* Hook. f. | 0.4 (3.3) | 0 |
| *Tradescantia fluminensis* Vell. | 25.9 (37.7) | 8.6 (24.3) |
| *Tradescantia zebrine* hort. ex Bosse | 0 | 0.2 (1.3) |
| *Vallesia glabra* (Cav.) Link | 0.2 (2.2) | 0 |
