# Supplementary material for: Limited natural regeneration of unique Scalesia forest following invasive plant removal in Galapagos
Source: PLoS One. 2021 Oct 13;16(10):e0258467. doi: 10.1371/journal.pone.0258467 (PMC8513895; doi:10.1371/journal.pone.0258467)
Supplement: S2 Table — (DOCX) [file pone.0258467.s004.docx]

**S4 Table:** Average and maximum growth of *S. pedunculata* saplings and young trees over time.

| **Observation period** | **Average growth (cm)** | **SD average growth** | **Max. growth (cm)** | **Daily average growth (cm)** | **Sample size (n)** |
| --- | --- | --- | --- | --- | --- |
| Apr 2015 – Jul 2015 | 46.7 | 36.2 | 220 | 0.51 | 146 |
| Jul 2015 – Feb 2016 | 62.5 | 41.3 | 206 | 0.29 | 60 |
| Feb 2016 – Aug 2016 | 66.6 | 56.2 | 239 | 0.35 | 46 |
| Aug 2016 – Mar 2017 | 30.5 | 38.3 | 111 | 0.14 | 45 |
| Mar 2017 – Jan 2018 | 49.4 | 49.1 | 119 | 0.18 | 30 |
| Jan 2018 – Apr 2019 | 33.0 | 62.9 | 165 | 0.07 | 21 |
| Apr 2019 – Mar 2020 | 32.6 | 56.0 | 110 | 0.10 | 13 |
| Apr 2015 – Mar 2020 | 457.6 | 161.2 | 733 | 0.25 | 13 |
